# Perceptions and Experiences of the COVID-19 Pandemic amongst Frontline Nurses and Their Relatives in France in Six Paradoxes: A Qualitative Study

**DOI:** 10.3390/ijerph18136977

**Published:** 2021-06-29

**Authors:** Stephanie Chandler-Jeanville, Rita Georges Nohra, Valerie Loizeau, Corinne Lartigue-Malgouyres, Roger Zintchem, David Naudin, Monique Rothan-Tondeur

**Affiliations:** 1 Nursing Sciences Research Chair, Laboratory Educations and Health Practices, Université Sorbonne Paris Nord, (UR 3412), UFR SMBH, 93017 Bobigny, France; ritag.nohra@gmail.com (R.G.N.); loizeauvalerie@gmail.com (V.L.); corinne.lartigue@aphp.fr (C.L.-M.); roger.zintchem@aphp.fr (R.Z.); david.naudin@aphp.fr (D.N.); rothan-tondeur@univ-paris13.fr (M.R.-T.); 2 Nursing Sciences Research Chair, Assistance Publique-Hôpitaux de Paris, 75005 Paris, France; 3 Anesthesia Department, Assistance Publique Hôpitaux de Paris, Hôpitaux Universitaires Paris Seine-Saint-Denis, Hôpital Avicenne, 93000 Bobigny, France; 4 Hôtel-Dieu de France Hospital, Alfred Naccache Boulevard, Beirut 166830, Lebanon; 5 Faculty of Public Health, Branch II, Lebanese University, Fanar 248199, Lebanon; 6 Nursing Care Management Department, Hôpital Poissy Saint-Germain-en-Laye, 78300 Poissy, France; 7 Avicenne-Jean Verdier Nursing Training Institute, Centre de la Formation et du Développement des Compétences, Assistance Publique-Hôpitaux de Paris, 93000 Bobigny, France; 8 Centre de la Formation et du Développement des Compétences (CFDC), Assistance Publique-Hôpitaux de Paris, 75571 Paris, France

**Keywords:** COVID-19, frontline nurses, families, paradoxes, well-being, France

## Abstract

Due to their frontline position to fight the coronavirus disease 2019 (COVID-19), the professional and personal life of nurses was severely disrupted. To understand and describe their lived experiences and perceptions during the pandemic’s first wave in France, we interviewed 49 nurses, including 16 nursing students, and 48 of their family members from June to July 2020. Using a purposeful sampling, the semi-structured interviews were scripted according to Abric’s method with probing questions. The interview analysis led to the identification of six paradoxical perceptions concerning the pandemic’s consequences: the Silence Paradox, the Hero Paradox, the Workforce Paradox, the Learning Paradox, the Symbolic Exchange Paradox, and the Uncertainty Paradox. However, despite different experiences, the nurses perceived their frontline position both as a burden jeopardizing their safety and well-being and as a spotlight of nurses’ tough working conditions. Indeed, because they were in the frontline position, nurses and nursing students were psychologically vulnerable, even more so when they felt alone and inadequately protected. Besides, their families were vulnerable too, as they were also exposed to the consequences of the nurses’ frontline engagement. Thus, to preserve their safety and well-being, institutions should also provide them with better organizational support and inclusive leadership, without neglecting their families.

## 1. Introduction

Since its inception, as of 4 May 2021, the new coronavirus disease (COVID-19) has affected worldwide 153,187,889 persons, including 3,209,109 deaths reported to the World Health Organization (WHO) [1]. In France, there have been 3,390,070 confirmed cases with 81,226 deaths [1]. Because of its high contagiousness, facing COVID-19 represents a double challenge for healthcare structures. They had to quickly reorganize their services to face the massive influx of patients, by increasing their bed capacity by different interventions [2].

Meanwhile, they also had to protect their workforce from being infected. However, within this pandemic context, pursuing these two goals was tough. Indeed, healthcare workers (HCW), including nurses, were confronted with structural problems, as they had to handle critical shortages of drugs, ventilators and Personal Protective Equipment (PPE) [3]. Thus, as they worked frontline to take care of infected people without the required protection, nurses were particularly exposed to the virus. Indeed, nurses were the most frequently infected among all HCW [4], and 2200 of them have died since the outbreak’s start [5]. Besides, previous studies, relating to other epidemics like Ebola [6], have shown that healthcare workers (HCW) and their families may be stigmatized to the point of physical or verbal abuse.

Because of their frontline position, nurses were more exposed to the physical and social consequences of the virus but they were also more at risk of developing severe emotional distress (i.e., depression, anxiety) as they were confronted both with an increased work stress and the high lethality of this disease [7,8]. These different elements had also consequences on their personal life and well-being, which have been poorly explored in-depth [9]. However, to our knowledge, no study has focused on the experiences and perceptions of frontline nurses’ relatives. Yet, families have been identified as resourceful for helping HCW to cope with the pandemic’s consequences [7]. Thereby, we sought to elucidate the consequences of nurses’ frontline position on their personal and social life, and to compare them with nurses’ own relatives.

Thus, to have a better understanding of the consequences of frontline position during this period, we needed to investigate those who have lived and experienced it, i.e., frontline nurses, nursing students, and their families [10]. Therefore, with this phenomenological study, we aimed to understand and describe frontline nurses, nursing students, and their family members’ lived experiences and perceptions of the COVID-19 pandemic.

## 2. Materials and Methods

For this research, we used a descriptive phenomenological approach, which according to Husserl, focuses on the study of a phenomena “as perceived by human consciousness” [11].

This qualitative study was conducted from June to July 2020, just after the end of the first lockdown in France, through video-conferencing semi-structured interviews.

In order to meet the objectives of this study, we interviewed a dyad each composed of a nurse or nursing student who worked in France in units receiving COVID-19 patients and one of their relatives aged over 18 years old. We used a purposeful sampling, and, to ensure diversity in the experiences collected, types of nurses and job setting were considered. The first recruited were known by the research team. The following were approached through the snowball method. The sample size was completed when data saturation was reached, i.e., when no new topics were generated.

Eligible nurse or nursing student and one of their relatives received an information letter by email to set a date for the interview according to their availability. Confidentiality was assured by deidentifying the verbatim through the following pseudonymisation scheme: first letter of the interviewer’s first name—nurses’ job setting-COVID-19-related redeployment units. A (f) was attached at the end of the pseudonym when the data were related to a family member. All video recordings and verbatim were saved on three password-protected computers. The study was approved by the CER U-Paris University institutional review board (IRB 00012020-52). There was no funding source for this study.

This work was led by nurses, all members of the same research team (i.e., Nursing Sciences Research Chair of Sorbonne Paris Nord University). As most of them were involved in the fight against COVID-19, they wished to focus this study on nurses, in order to raise awareness about the consequences of their frontline engagement. Thus, six researchers (M.R.T., V.L., C.L.M, R.Z., D.N., and S.C.J.) conducted the semi-structured interviews, using the same interview script. As the research topic was sensitive, regular meetings were organized to enable the researchers to discuss their findings and share their experience with the other researchers to better cope with the toughest interviews. To report these findings, we followed the Standards for Reporting Qualitative Research guidelines [12].

The interviews were realized through video-conferencing with participants’ consent. The participants could choose the software (Skype^®^ or Zoom^®^) and be off screen, while they were interviewed sequentially. Baseline characteristics for nurses (age, marital status, years of work experience, type of COVID-19 unit where they worked during the health crisis) and for their relatives (age, nature of the relationship with the nurse, type of job) were asked at the start of the interview.

We developed an interview guide to explore the participants’ perceptions and experiences, based on Abric’s theory of central core [13]. This model was inspired by Moscovici’s work in social psychology, with the theory of social representations [14]. According to Abric’s approach, social representations provide guidelines for reading and understanding the world. Thus, representations allow behaviors and actions to be explained and also meaning to be given to practices [13]. We did not aim to define the social representations related to the pandemic in this study. However, we considered this approach accurate in limiting social desirability answers [15]. Indeed, it enables indirect questions to be asked by grasping the “mute zone”, where the participants can attribute certain ideas to others in order to reduce “normative pressure” [16]. Thus, probing questions, such as “What was your colleague’s feeling about this?” were used to explore the mute zone and enhance the depth of discussion. Potential sensitive issues could occur during the interviews. An information leaflet presenting psychological support platforms was sent by email at the end of each dyad’s interview to prevent secondary psychological harm.

No transcriptions have been realized since we used the Atlas Ti Scientific Software Development GmbH (Berlin, Germany), which enables the video files’ content to be analyzed directly [17]. The interviews and data analysis were in French. Only the selected quotations were translated into English by one researcher and back translated by another to ensure the right translation.

To ensure the results’ credibility and trustworthiness, these two researchers analyzed the interviews independently, before comparing their findings. They were then audited by a third senior researcher, expert in the use of qualitative methods in nursing research.

Thus, the interviews were subjected to axial coding using ATLAS.ti 7 and manually by two different researchers [18]. This method enabled recurring significant sentences and words to be highlighted then coded according to the strength of their occurrence. The codes were then organized according to the frequency within the corpus through a descriptive analysis, to highlight the characteristics of the people interviewed, their codes, their themes, and their perceptions.

Afterwards, the concordance or discordance of speeches between nurses and their relatives were studied through a comprehensive analysis. Finally, the two researchers carried out an interpretative analysis with a third senior researcher. The findings were then discussed and validated, which led to the definition of paradoxical experiences and perceptions as participants shared their experience of paradoxical situations during the pandemic. According to Parse, lived experiences are paradoxical, as “paradoxes are not opposites, but are dimensions of the same rhythm lived all-at-once where one is in the foreground and one is in the background” [19,20].

## 3. Results

Our sample consisted of 49 nurses, recruited from different hospitals in the Paris area, and 48 of their relatives. Only one relative refused to take part in the study at the last minute. Interviews lasted from 18 to 84 min (mean 42 06 min). Considering the nurses, 33 of them were certified and 16 were students, most of them in the last year of their nursing formation. Most of the nurses (39%) worked in the Intensive Care Unit (ICU) and 26% took care of the elderly (in nursing homes or in hospital). More than two-thirds of the interviewed relatives were their spouses, and more than one third (35%) have a health-related job.

The analysis resulted in six theme categories, which led to the definition of six paradoxes related to nurses, nursing students, and their families opposite feelings about their lived experiences during the pandemic, beyond their common shock. We present them alongside representative quotations. Following the aforementioned pseudonymisation pattern, we kept the initials of nurses’ job setting and of their COVID-19-related redeployment units as follows:

- RN for Registered Nurse, NS for Nursing Student, CRNA for Certified Registered Nurse Anesthetist, NM for Nurse Manager;
- GS for Geriatric Service, PACU for Post-Anesthesia Care Unit, ICU for Intensive Care Unit, PS for Pool Service, M for Medicine, NH: Nursing Home;
- a (f) was attached at the end of the pseudonym when the data were related to a family member.

### 3.1. The Silence Paradox

In this study, the first lockdown led participants to experience and perceive two opposite sound atmospheres, whether they were inside or outside the hospital.

Indeed, as nurses and nursing students were directly exposed to COVID-19, they were confronted with different forms of silence. There was a form of silence induced by the perceived omnipresence of death in the units:

“We were in our service but the atmosphere was so strange... It was as if death was there all the time in the service above.”

RN-GS

Besides, nurses were also confronted with another form of silence of their top managers, which was perceived as an upsetting lack of support and interest about their frontline-related issues:

“There was a lack of support and information from the management. A soldier needs his captain to be there to boost and encourage him.”

CRNA-PACU

As they faced the high lethality of this disease and feared for their relatives’ health and their own, most of the nurses chose to remain silent. Indeed, they refused to share their emotions with their families and concealed them, in order to protect them:

“I tried to talk to my husband about it, but as he’s not involved in healthcare, I know that he’s quite sensitive about it, so I didn’t want to worry him.”

NS-ICU

Thereby, nurses’ relatives experienced their silence, and felt helpless:

“She didn’t even talk about what was going on, and didn’t tell me what was going on at the hospital. She kept it to herself so I thought it must have been difficult and she wanted to protect us.”

RN-PS (f)

However, few nurses experienced a constrained silence, as they needed to express their feelings but they did not find the required resources, neither from psychologists nor from their managers.

At the opposite side of these perceived forms of silence, noisier elements accompanied nurses and their relatives’ lives, outside the hospital. Thus, they enjoyed the noise related to the evening handclapping campaign, as they perceived it as a proof of society support:

“However, at 8 pm, it was a bit like a meeting of all the neighbors and we applauded. The children: it was good for them to know that we were applauding for their parents.”

NMS-ICU(f)

Nevertheless, the noise coming from the continuous media reports about the pandemic was unwelcome. Indeed, nurses’ family members perceived this overload of information as a stressful experience, which increased their fears for them and for nurses:

“There was also an anxiety related to TV reports and I preferred to cut it off because it was too focused on the number of deaths per day…My spouse was requisitioned, and I was stressed by that.”

CRNAS-ICU (f)

### 3.2. The Hero Paradox

Nurses and their relatives perceived the nursing profession differently during the outbreak.

Most of the nurses did not consider themselves as heroes because they worked during the outbreak. They considered it as their duty. However, they were proud of what they did, as they kept working despite their fears and their medical conditions (cancer, asthma):

“I was stressed about my health because people like me [with a history of cancer] were vulnerable so I wondered if I was doing something stupid on the way to work. Afterwards, I thought yes I was more at risk, but that is my job after all.”

CRNA-PACU

Nurses’ pride also came from their awareness of their professional skills’ efficiency, as some of them faced unusual care:

“Through this crisis, you discover yourself as a professional with knowledge and skills. A feeling of pride in everything I’ve accomplished, reassured to see that I could do things that I thought were insurmountable, pride in myself, pride in a profession where people have unlimited self-sacrifice.”

CRNA-ICU

Meanwhile, nurses’ relatives considered nurses and nursing students as heroes and were proud of them because they put their life at stake in caring for others despite their exhaustion and their difficult working conditions.

“Afterwards, this outbreak showed me even more how bad things are at hospital: all the problems nurses usually experience have been multiplied tenfold.”

RN-M (f)

However, nurses and their relatives were disappointed and angry by the public authorities’ lack of recognition for nurses’ engagement despite their heroisation:

“We were angry, we don’t even talk about money: we just talk about a thank you, it would have been nice if the management had simply thanked us.”

NM-ICU

Thus, they considered this crisis as an opportunity to raise society’s awareness about the French healthcare system’s failures and to promote a better recognition of the nursing profession. However, some nurses were quite skeptical about the heroization effects on society, as they and their families were confronted with stigmatization.

“For me, nurses are not recognized for their commitment, their efforts, their daily risks. In my opinion, it is not a medal on July 14th that will give them the recognition they deserve. They must have the salaries they need…There are years of studies. We have to give them the means to work well. It’s their lives they are putting at stake.”

RN-PS (f)

“We were considered a bit pestiferous, people were afraid of us, afraid that we might contaminate them, afraid that we might be contagious.”

NS-M

### 3.3. The Workforce Paradox

Nurses reported that their working conditions were better if they worked in acute services as they were overstaffed, whereas nurses from other departments were confronted by a nursing shortfall.

Indeed, nurses who worked in geriatrics or nursing homes suffered from a usual lack of personnel but it worsened during the crisis, and they had to work overtime, even when sick. Moreover, some nurses feared becoming infected and refused to take care of COVID-19 patients, which led to increased workload for the remained colleagues. Thus, nurses were angry about these working conditions, and considered they resulted from cost saving healthcare policies.

“I think the hospital is as sick as its patients… We are asked to take upon ourselves. It can be exhausting. It is true that sometimes when you are in a department with very few staff, you can talk about suffering.”

RN-GS

“My colleagues were very worried about the virus. Actually, many of them, who considered themselves to be at risk, refused to work. A lot of nursing assistants refused to enter rooms, for example to give breakfast to patients in the COVID-ward, obliging me, my fellow trainees, and other nurses to do this work for them, which added to the workload.”

NS-GS

On the opposite side, COVID-19 units were often overstaffed, which contributed to improve the quality of care by enabling nurses to organize better. Moreover, some nurses reported that they were really supportive of each other, which improved teamwork and the cohesion between team members. This collective power helped them to face the adverse events occurring during this crisis. Because of this, some ICU nurses were reluctant to have work breaks, even enjoying working during this period.

“There was a very good atmosphere between colleagues. There was a kind of working cohesion where everyone helped each other. I was able to rediscover a working atmosphere that I had known a long time ago and which had disappeared with the budget cuts. It gave me the strength to tell myself that I wasn’t alone.”

CRNA-ICU

### 3.4. The Learning Paradox

Because of the massive influx of patients related to the pandemic, nursing students, in initial or continuing training, were requisitioned to strengthen frontline teams. This training break was perceived and experienced differently by the students, as to whether they felt useful or not during this period.

Indeed, most students wanted to be useful but they were also anxious about the skills expected from them:

“My fellow nursing students were like me, not very reassured, as their position was unclear. We didn’t know if we were considered as a nursing student or as a nurse assistant.”

NS-GS

Thus, when nursing students felt poorly tutored and integrated with the staff, as if they were more used than useful, some of them could be considered as collateral victims of the virus, as one nursing teacher reported it:

“I had the impression that the students were taken hostage and were unable to follow their training. And that is the collateral effects of COVID because they experienced it very badly and me too because it was necessary to support them, to accompany them, and it was complicated to manage.”

NM-ICU

However, some students considered this experience a professional opportunity, as they could deepen their nursing skills and also obtain new ones. Thus, these nursing students estimated that their training had not been altered by this period:

“It was an opportunity for me to work under such conditions. And as a student, the fact of being positioned both as a support for the team and as a student was formative and helped me getting new skills: autonomy, taking initiatives.”

NS-ICU

This period was also valuable for some certified nurses, who learnt new skills from colleagues, and wished to extend them further by obtaining new degrees.

“It was an opportunity for me to work under such conditions. It helped me getting new skills.”

NS-ICU

### 3.5. The Symbolic Exchange Paradox

As nurses and nursing students were engaged frontline, they experienced sacrifices as they were robbed of a part of their personal and professional life. However, in exchange, this position induced some gains, as they felt fully supported by their family and society.

Concerning their professional life, nurses and nursing students felt powerless and frustrated as they considered that COVID-19 had stolen essential features of their job, especially the ones related to end of life care:

“Some residents died, they were completely suffocating and nothing could be done: it was difficult…These images chased me for a while.”

RN-NH

Indeed, some of them pointed out that the nurse-patient relationship was deeply disrupted by the protective measures (i.e., wearing of PPE outfits) and their consequences (i.e., limited contact and time spent with the patients, even when they were dying):

“The care was different because of the protective outfits we had to put on. It’s a different approach of care, as everything that has to do with touch or even smiling, we didn’t have that anymore or at least we limited it.”

NS-GS

Moreover, nurses found the ban of patients’ visits a bad experience.

Thus, nurses also considered that patients families’ grieving process was robbed by the virus, as they were not allowed to see their relative’s body:

“The families could not see the people who had died, but in addition there were no religious rites and they were only washed with water.”

RN-M

Besides, nurses experienced in a bad way the disruption of their professional values through the ethical dilemma induced by patients’ prioritisation, concerning mostly the elderly. Some nurses found this process difficult to accept, because they considered it as loss of chance for the excluded patients:

“It was extremely violent to apply Do Not Resuscitate choices for people in their 70s, without risk factors. It’s complicated to tell them that there is no place in ICU for them.”

NP-M

Meanwhile, some nurses reported that the COVID-19 had also stolen their health, as they had to work regardless of their exhaustion and of their health conditions, which made them angry, especially in nursing homes:

“I was infected three months ago and I still have symptoms of COVID. Total exhaustion with asthma-like respiratory discomfort, muscular pain: I currently have a post-COVID syndrome, which is disabling. I can’t do a lot of activities, because otherwise I have to sleep and I’m exhausted. I’m angry because I was sent to work, even when I was sick. And now I’m paying the price.”

RN-NH

Concerning nurses’ family life, this was disturbed by their increased job engagement, which made it difficult for them to keep the balance between their family and professional life, causing them to feel guilty and angry. However, nurses were grateful for their spouses’ support, as they were more involved in their household life management, even though it was harder when a nurse’s spouse was also an HCW:

“For two months we didn’t live for the family, we lived around COVID.”

CRNA-PACU

“I still had a feeling of guilt because even if you come home, you’re not too available to do anything else. Helping the children in their homework, even on days off, I couldn’t do it.”

CRNA-ICU

Moreover, as they feared spreading the virus, nurses had limited physical contact with their relatives, even leaving their home. Thus, this period was emotionally tough for all participants, especially when children were involved. Some spouses felt deprived of the nurses’ presence at home because of their work’s engagement, and their relationship was affected:

“When I came home, the children didn’t want to come near me, they even gave me the name Mummy COVID and said: ‘No kisses Mummy because you’re COVID!’ So there was this distance between my family and me because I had to protect them.”

NS-NH

However, despite all these negative effects, the outbreak was also a period of donations and gains. Indeed, nurses and their families took advantage of the lockdown to question their priorities and to strengthen ties, as they enjoyed spending time together (e.g., cooking, baking, playing games). They were also deeply grateful for their friends and other family members’ support through this tough period. Besides, nurses appreciated the gifts coming from the entire society to support them:

“We felt supported during this whole period by the outside, by the people who clapped every night, by the people who delivered meals, by the drawings. This multiple support carried us, as perhaps unconsciously, it has helped us to better overcome this crisis, to get through it. There was a real surge of solidarity.”

RN-GS

### 3.6. The Uncertainty Paradox

Overall, participants perceived their own vulnerability, which they expressed through the certainties and uncertainties acquired through the pandemic management.

Concerning their family life, nurses and nursing students were deeply uncertain and anxious about how to avoid spreading the virus, when they came back home from work. Indeed, nurses and nursing students were deeply uncertain about their protection equipment’s effectiveness and use. It led them to become anxious and fearful of spreading the virus to their family members, because they were uncertain of the required measures to avoid spreading it, as they came back home from work:

“I was stressed to bring the virus home. So, I tried to set a strategy to avoid that…I’ve had patients who got sick because their spouse is a healthcare worker, one of them died and I didn’t want that to happen to me.”

CRNA-ICU

Besides, nurses also experienced uncertainties about their practices, as they were confronted to hygienic changing protocols, drugs and ventilator shortages and the doctors’ doubts about the medical treatment. Because of these elements, nurses and nursing students felt insecure and confused, which emphasized their fears and anxiety:

“We couldn’t protect ourselves as we should have. As if we were thrown into the lion’s den. Because in the beginning, the protocol was to put on masks only if patients coughed. Then afterwards, there were some who didn’t cough but who got COVID, so we were told to put on surgical masks, and then if they tested positive, to put on N95 masks and glasses…We didn’t feel protected by the protocols.”

NS-M

However, through this crisis, nurses also acquired some certainties through the poor PPE shortages management, which, for them and their families, was related to hospitals’ unpreparedness. Besides, some nurses, especially in nursing homes, felt devalued as they noticed COVID-19 units had more PPE than other units. This imbalance between services angered them, as they would have wished for more safety and better working conditions:

“Nurses in non-acute services did not feel they were treated in the same way as those working in COVID units. They lived this situation very badly and felt forgotten by the management.”

RN-ICU

Thus, some nurses were certain that they wanted to quit their job after the crisis, while others gained confidence and wanted to keep working as a nurse. Indeed, of the 55 nurses interviewed (including relatives): four nurses (7%) expressed their desire to leave the nursing profession, four other nurses (7%) would have left the nursing profession if they had not already been engaged in a career evolution and five nurses (9%) are considering leaving their establishment following this health crisis. Thus, nearly a quarter of the nurses (23%) admit that they are considering a change of career path. However, this might be an underestimation, as other nurses complained about the poor management they had experienced.

“I’m thinking of changing my profession to go towards what is human, such as sophrology and relaxation, because I have the impression that there is no longer any humanity, either with the patients or the staff.”

RN-NH

## 4. Discussion

Since the coronavirus disease’s onset, nurses have been at the forefront, taking care of infected patients in tough circumstances, at their own physical (e.g., infection risk, exhaustion) and psychological risks (e.g., severe emotional distress from anxiety to burnout). Thus, their family and social life have been deeply disrupted. With this study, we aimed to understand and describe frontline nurses, nursing students, and their family members’ lived experiences and perceptions of the COVID-19 pandemic. The qualitative design of this phenomenological research enabled nurses and their relatives to express their paradoxical experiences and perceptions related to the COVID-19 first wave and its consequences on their personal, professional, and social life. However, our findings also highlight that both of them were collateral victims of the pandemic because of nurses’ frontline position.

Despite their heroization and the militaristic language used by society and public authorities to describe their actions during this health crisis, our findings suggest that nurses did not consider themselves either heroes or soldiers.

Indeed, they experienced psychological vulnerability, as they felt strained physically and emotionally by their relentless exposure to the virus consequences. Thus, as in previous studies [21], nurses and nursing students were driven by their sense of responsibility to work frontline but the challenging and harmful working conditions (i.e., PPE and medical devices shortages) jeopardized their own and the patients’ safety. Thus, nurses felt overexposed to the virus and were vulnerable to death anxiety, which they experienced through the high COVID-19 mortality rate or their inability to help patients as in previous researches [22]. Our results also highlighted nurses’ perceptions of other death anxiety factors, as the avoidable deaths, the rapid health declines leading to death, and the lack of human dignity for the dying and the deceased patients (e.g., Symbolic Exchange and Uncertainty Paradoxes) [22]. Besides, nurses’ vulnerability was also induced by their difficulties in providing care in this context [23,24,25] which disrupted their professional core values as they faced ethical dilemmas through patients’ prioritization and the lack of human dignity surrounding patients’ deaths [24,26,27]. Indeed, nurses were shocked by some of COVID-19 patients’ deaths as they considered they would have been avoidable if there had been no patient prioritization [24]. These different situations strengthened nurses’ death anxiety and weakened their mental health, as their frontline position reminded them of both their own vulnerability and that of their families. Nursing students felt also vulnerable, at the opposite side of another study [28], but lesser than registered nurses.

Thus, being in the frontline position deeply undermined nurses’ professional self, which led most of them to be more likely to demonstrate self-sacrifice (i.e., when they were asked to work regardless of their health and exhaustion) [29]. A previous research has shown that self-sacrifice is an intrinsic coping strategy for nurses to overcome tough situations, when they do not feel sufficiently supported [29]. Therefore, as their job engagement during the pandemic came with a personal cost, nurses required their skills and efforts to be really acknowledged. However, in our study, they were praised by their relatives, but less by their institutions [30], as they received poor gratitude for their engagement and their expertise was not requested in the anti-epidemic strategies decision-making (e.g., the Silence Paradox, the Symbolic Exchange Paradox, the Uncertainty Paradox) [31]. Thus, despite the fact that they represent the most important healthcare workforce, most nurses felt abandoned and complained about their top managers’ lack of inclusive leadership [25]. Inclusive leadership refers to “leaders who exhibit visibility, accessibility, and availability in their interactions with followers” [25]. This was perceived by French nurses as an additional lack of recognition of the nursing profession by their managers and the health authorities [30], as other nurses worldwide [32,33], which angered them.

Their families, who also endured psychological vulnerability, shared this anger. Indeed, our study stated that being related to a frontline nurse was a cause of fears and anxiety, as their families were afraid that nurses became infected being more exposed to the virus. Thus, through their perceptions (e.g., the Silence Paradox), nurses’ families shared their vulnerability to infodemic [34], an “over-abundance of information—some accurate and some not”, which could lead to their anxiety [34]. Indeed, the media coverage (e.g., the shortage of PPE and deaths tolls reports) added to the misinformation contributing to increasing the fears of nurses’ relatives. To avoid this infodemic anxiety, it is crucial to set up educational online programs, to provide reliable and accessible information available for everyone.

Thus, families should also be supported because frontline status could induce parenting troubles and stigma, as reported in previous studies [8,35].

Our findings also suggest that, in the opposite position of another research [36], some of the nurses interviewed in this study were not happy during the first coronavirus wave because of their frontline position. Indeed, owing to the Job Demands-Resources model [32,36], nurses were confronted with higher job demands (e.g., heavy workload, overtime work, ethical dilemmas) as identified previously [32], whereas their job resources were low (e.g., lacks of organizational support and inclusive leadership). Nurses considered this imbalance was related to failures of organizational support resulting from years of cost savings in healthcare, which angered and upset both them and their families [37]. Since the pandemic’s first wave, others nurses have reported the same anger and disappointment [38,39,40].

Thus, in our study, the more nurses felt alone and unprotected by their institution, the more they wanted to quit their jobs; almost a quarter of nurses (23%) are considering leaving their hospital or profession after this crisis. In previous studies, nurses expressed the same or opposite feelings [21,26].

**Figure 1:**
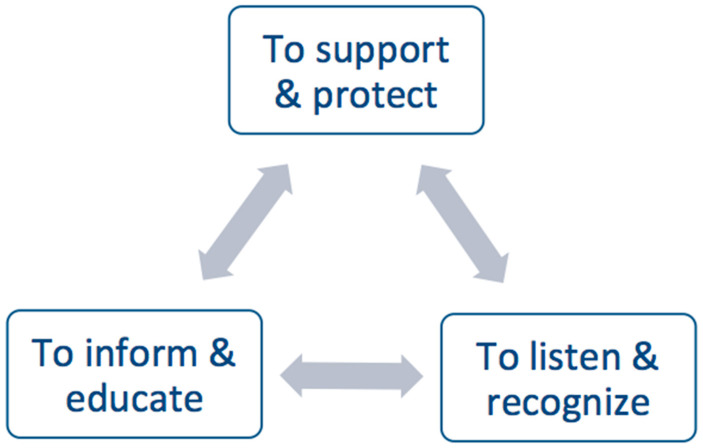
Strategies to preserve the well-being of frontline nursing workforce and their families.

As in previous research, our findings confirm that, as they worked frontline during the COVID-19 outbreak, nurses were physically and emotionally drained and vulnerable [41]. Thus, their well-being was threatened and multidimensional supportive responses should be provided to preserve this workforce in particular [41]. Thus, healthcare institutions should alleviate the perceived low job resources by improving inclusive leadership and organizational support through different strategies, which should also include their families as shown in Figure 1 [25]. Thus, institution managers should better recognize nursing issues [25,42], enhance frontline nurses’ job engagement through gratitude expressions and rewards, and organize the provision of tailored psychological support to help nurses, nursing students, and their families to manage their anxiety [22,36]. It is also crucial to provide nurses’ families reliable and comprehensive information through educational online programs [43]. This intervention could be based on the crisis education model developed by the Health Education and Practices Laboratory (LEPS UR 3412) of the Sorbonne Paris Nord University [44].

### Strengths and Limitations of the Study

One of the strengths of our study is the fact that it focused on frontline nurses and their families. Another strength of our work is that we recruited frontline nurses, including nursing students, from different settings and services with varying sociodemographic characteristics (e.g., different ages and length of work experience) despite the COVID-19 pandemic.

Therefore, we were able to establish a broad picture of the consequences of the nurses’ frontline position on them and their families in France, which has not been studied yet through qualitative research. Indeed, despite the qualitative design of this study, the sample size was large enough to explore and understand nurses’ and their families’ perceptions. A limitation of this study was that all the interviews were conducted through video-conferencing after the first wave, thus the feelings and perceptions of the respondents could have been lowered. Yet, some nurses were emotional, as this study dealt with sensitive issues. Thus, it was clearly stated that they could stop the interview at any moment. Another limitation of our study may be seen in the study design, which can compromise the results’ transferability and generalization. Further research is needed in other countries, to grasp the lived experiences of other nurses and their families.

## 5. Conclusions

World Health Organization (WHO) declared 2020 the “Year of the Nurse and Midwife”. It should have been a time to celebrate nursing profession but because of the COVID-19 pandemic’s hardship, nurses had to show their professional strengths and meanwhile healthcare systems were undermined.

However, as nurses are the most important healthcare workforces, their increased frontline engagement impacted them in their personal, professional, and social life. Thus, our findings provide useful insights in describing and exploring the experiences and perceptions of nurses, nursing students, and their families during this period in France. The frontline position appears mostly as a factor of suffering, as nurses and nursing students were emotionally and physically strained. Their vulnerability was reinforced when they felt abandoned and inadequately supported by the top managers. After one year of pandemic pressure, these findings remain accurate, as nurses’ working conditions worldwide are perceived to be unsatisfactory to some of them, who still feel poorly rewarded and supported. Therefore, institutions should address their specific concerns (i.e., better recognition, better organizational support, and inclusive leadership) to preserve nurses’ job engagement and resilience. However, our findings highlighted that their families should also have been supported, as they were exposed to the consequences of the nurses’ frontline engagement (i.e., nurses’ absence, parenting troubles, and infodemic).

Through this pandemic, participants’ perceptions about the nursing profession have been questioned, despite their heroization. After one year of pandemic pressure, our findings remain accurate, as nurses’ working conditions worldwide are perceived to be unsatisfactory by some of them, who still felt poorly rewarded and supported by their institutions.

## Data Availability

The data presented in this study are available on request from the corresponding author, and after receiving permission from Nursing Sciences Research Chair.
